# Occupational exposure to respirable crystalline silica in municipal household waste collection and road cleaning workers

**DOI:** 10.1038/s41598-021-92809-5

**Published:** 2021-06-28

**Authors:** Boowook Kim, Eunyoung Kim, Wonseok Cha, Jungah Shin, Byung-Soon Choi, Daeho Kim, Miyeon Kim, Wonyang Kang, Sungwon Choi

**Affiliations:** Institute of Occupation and Environment, Korea Workers’ Compensation and Welfare Service, 478, Munemi-ro, Incheon, 21417 Korea

**Keywords:** Environmental sciences, Health occupations

## Abstract

Despite the increase in the number of cases among South Korean sanitation workers, lung cancer as a result of exposure to occupational carcinogen has not been sufficiently investigated. This study aimed to identify exposure levels of sanitation workers to respirable crystalline silica (RCS) for various tasks and factors that affect individual RCS exposure. Exposure to RCS was assessed for 90 sanitation workers from seven companies. The obtained geometric mean value of the RCS was 2.6 µg m^–3^, which is a similar level to recommendations set by California’s Office of Environmental Health Hazard Assessment's Recommended Exposure Limit. Meanwhile, coal briquette ash (CBA) collectors exhibited the highest RCS concentration (24 µg m^–3^), followed by road cleaning workers who used a blower, municipal household waste collectors, sweepers, and drivers (p < 0.05). Additionally, when the ANOVA was conducted, statistically significant differences were observed in RCS concentrations among various factors such as job task, season, employment type and city scale. Our study confirmed that sanitation workers who work outdoors could be exposed to RCS. Due to the possibility of exposure to high RCS concentrations, special attention should be paid to the collection of used CBA and road cleaning involving the use of a blower.

## Introduction

In many countries regular waste collection is conducted as the interest in the recycling and safe management of municipal household waste (MHW) has increased over the past few decades. However, workers who collect waste face an increased risk of musculoskeletal and various respiratory diseases, through exposure to dust, bioaerosols, and diesel engine emissions^1^.

As of 2016, the total number of sanitation workers in South Korea was approximately 33,950^2^. Diesel particulate matter (DPM) is the major toxic material exposed to sanitation workers. The International Agency for Research on Cancer (IARC) classified this as a Group 2A substance, which is “probably carcinogenic to humans”, and then in 2012 reclassified this as a Group 1 substance, which is “carcinogenic to humans”^3^.

In South Korea, compensation claims of sanitation workers due to the occurrence of lung cancer has been increasing, and several work-related assessments (epidemiological surveys) have been conducted as a result. The number of cases where compensation claims are accepted with public compensation has also been increasing^4^. As such, the Korean Government has significant interest in the health issues of sanitation workers, which are gradually becoming a social concern as media reports increase^2^.

Exposure to DPM emitted from waste collection and other vehicles is inevitable for sanitation workers. As DPM is a Group 1 carcinogen designated by IARC, it is important to assess and decrease the exposure levels for workers. Therefore, previous studies have often focused on the DPM exposure of MHWworkers^5–10^.

Sanitation workers, however, can be exposed to respirable crystalline silica (RCS), which is also designated as a carcinogen by IARC^11^, however, studies on RCS exposure are rare.

In particular, RCS is a major carcinogen with a binding occupational exposure limit value (BOELV) established by the European Union. Therefore, it is a substance that requires active intervention to reduce worker exposure in the working environment^12–14^.

RCS can cause various respiratory diseases, such as lung cancer, silicosis, and chronic obstructive pulmonary disease (COPD), and is also associated with autoimmune diseases, such as rheumatoid arthritis and gastric cancer^15,16^. The occupational exposure limit (OEL) for workers is set at 0.1 mg m^–3^ in the EU^13^, 0.05 mg m^–3^ in South Korea^4^, and 0.025 mg m^–3^ in American Conference of Governmental Industrial Hygienists (ACGIH)^4^. Meanwhile, the California Office of Environmental Health Hazard Assessment (OEHHA) has set levels at 3 µg m^–3^, which is a chronic reference exposure level (REL) for the general public^17^. Recently, South Africa emphasized the need to establish non-occupational silica criteria while expressing concerns over health risks caused by exposure to silica in the atmosphere^18^. A recent study by Vacek et al.^19^ showed that workers with longer exposures at lower concentrations had a higher risk of silicosis than workers with the same cumulative exposure who worked for a shorter time at higher concentrations.

The representative tasks of sanitation workers are divided into road cleaning (RC; street cleaning) and MHW collection, while in Korea, MHW is classified into three types: solid waste, food waste, and recyclable materials such as plastic, paper, cans, clothes and bottles^7^.

When sanitation workers perform RC, they usually pick-up trash with tongs or perform sweeping. Sometimes they use air blowers mounted on their back while walking along the street to push the sand, soil, and fallen leaves out of the street. In this process, the sand and soil on the ground could scatter into the air, and the RC workers may be exposed to quartz, which is the most common form of crystalline silica within soil.

In addition, in East Asia, including South Korea, China, and Japan, as well as additional countries in Central Asia, such as Mongolia and Kyrgyzstan, coal briquettes are used as fuel for home heating and in restaurants for roasting. Currently, most households in South Korea use natural gas for heating, except for some low-socio-economic households and rural areas. In 1988, during the Seoul Olympic Games, a large amount of coal briquettes were used as 78% of Korean households relied on these as fuel for heating during that time.

Coal briquettes are fossil fuels made by compressing coal powder in specific molds and are used to obtain heat through combustion.

However, coal contains 3–7% quartz, and due to the evaporation of carbon components and moisture, the burnt coal briquettes turn pale pink and become loose coal briquette ash (CBA), although their shape is maintained. It appears that quartz remains the main component of this CBA. As such, sanitation workers can be exposed to quartz contained in the CBA during collection and handling. However, data collection on the quartz exposure level of sanitation workers is challenging^20^.

In addition, previous studies^5–10^ have focused on MHW worker exposure to dust, bioaerosol and diesel exhaust during general, food, and recycling waste collection operations, but the sanitation workers perform MHW collection in addition to road cleaning and CBA collection.

The work content and workload may also vary among workers directly employed by local governments and those hired by subcontractors. Exposure to dust and RCS can differ depending on various factors, including city size and season.

Therefore, the purpose of this study was to identify the following: (1) RCS, respirable dust (RD), and total dust (TD) exposure of MHW and RC workers, (2) correlations among these factors, and (3) factors affecting individual RCS exposure.

## Methods

### Task description by companies

The investigated scenario was as follows: Companies A, B, and E collect the MHW. One team of two MHW workers and a driver collects waste while traveling in the specified area. The workers move by hanging onto the back of a truck and running for short distances. The collected waste is thrown to the press roll space at the rear end of the truck and moved into the truck container while being pressed and crushed. While the majority of waste is contained in plastic bags, CBA can also be found within a plastic bag adjacent to 4–6 units or stacked on the roadside without plastic bags. Once pressed, the CBA is crushed, and the dust is scattered into the air.

Company A and Company B collected approximately 100 used coal briquettes, while Company E collected 300 on the measurement day.

In the local government where Companies B and E are located, CBA is collected and buried with general waste. In contrast, in the local government where Company D is located, CBA is collected separately and sent to coal briquette recycling facilities where it is used as an industrial resource. Thus, in Company D, a team dedicated to the collection of CBA was operating and collected CBA contained in plastic bags using an ordinary truck without a press roll while visiting sites in the area for approximately four hours. They manually removed the CBA from the plastic bags in a designated location and placed this onto a conveyor belt to allow it to be stored in a container because only CBA can be inserted into the recycling facility. The task to transfer CBA into a container took approximately 40 min, during which time the CBA was crushed, and the resulting dust was scattered into the air in large quantities.

Street cleaners pick up trash using tongs and sweep foreign matters, such as sand, soil, and fallen leaves while walking alongside the road or sidewalk.

The tasks conducted at Company C include leaf blowing on the sidewalk using an air blower for 20–30 min/day, and then performing typical street cleaner tasks for the remainder of the day.

Company F is the street cleaner of a small local city. Due to the hilly topography, the company applies sand and calcium chloride to the road in early winter to prevent cars from skidding due to snow and rain. Then, sand and calcium chloride are removed using air blowers and sweeping for approximately 40 days in the spring when snowfall stops.

Company G also measured the “dedicated CBA collector”. The difference between Company D's “dedicated CBA collector” is that Company G manually loads (throws) briquettes on the roadside into truck. Then, the collected CBA are automatically removed from the truck into unused rice fields.

**Figure 1:**
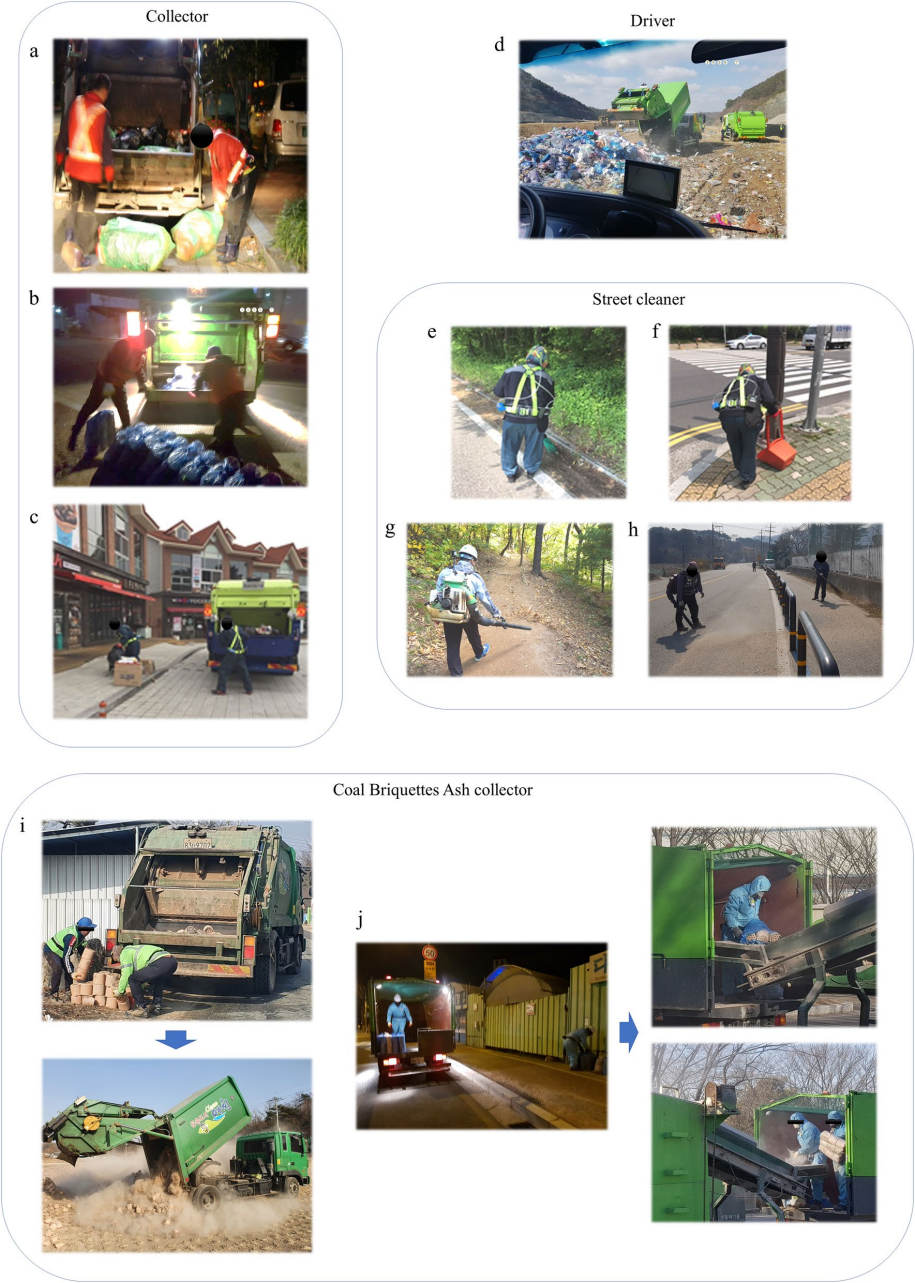
Tasks of the sanitation workers in this study. (**a**) loading general garbage in a truck, b: Loading CBA in a truck, (**c**) loading recyclable garbage in a truck, (**d**) driver of garbage truck, (**e**) sweeping the road, (**f**) Picking up trash, g: air blowing fallen leaves, (**b**) air blowing road sand, (**i**) the CBA is manually loaded onto the truck, and the truck automatically unloaded the CBA. (**j**) Manually loading and unloading briquettes into a truck.

In contrast, in this study, there were as few as four dedicated CBA collectors. This is because many local governments do not operate dedicated CBA jobs, and CBA workers were difficult to recruit as research subjects. Table 1 shows the summary of study companies, job task, and number of RCS samples. Figure 1 illustrates the various tasks of the sanitation workers used in this study.

**Table 1:** Summary of study companies, job task, and number of respirable crystalline silica samples.

Company	Sampling date	Measurement target	N^a^	City	City scale	Employment type
A	17.5.11	MHW^b^ (recyclable)	12	Ulsan	Large	Directly
		Driver	5			
		Street cleaner	13			
B	17.10.19	MHW (normal & food)	18	Jinju	Small	Employed by subcontractors
		Driver	4			
		Street cleaner	8			
C	18.10.24	Street cleaner (fallen leaves blowing)	4	Incheon	Large	Directly
		Street cleaner	1			
D	19.2.1	Used coal briquette ash collecting and Loading	2	Bucheon	Small	Employed by subcontractors
E	19.2.11	MHW (normal)	12	Gwangju	Large	Employed by subcontractors
F	19.4.12	Street cleaner (road dust blowing)	9	Goesan	Small	Directly
G	21.1.13	Used coal briquette ash collecting	2	Yecheon	Small	Directly
Total			90

### Exposure group selection and evaluation of the exposure levels of TD, RD, and RCS

Seven companies were selected as our study targets from various areas in South Korea. The detailed job tasks (occupations) were MHW workers, collection vehicle drivers, sweepers (fallen leaves and sand blowing), and CBA collectors.

The total dust (TD), respirable dust (RD)^21^, and RCS concentrations exposed to a worker during the daily working hours were evaluated based on NIOSH 0500^22^, 0600^21^, and 7500^23^ methods, respectively. TD and RD were collected at the same time of day using the GilAir Plus personal pump and dual port manifold. TD was collected at a flow rate of 1.5 lpm using a three-piece cassette (SKC Cat No. 225-2LF) equipped with a PVC filter paper (SKC Cat No. 225-5-7) with a 37 mm diameter and a 5 µm-pore size. RD was also collected at 2.5 lpm using an aluminum cyclone (SKC Cat No. 225-01-02) equipped with a PVC filter paper, which was stored in a desiccator for 24 h before and after measurements to remove moisture. Then, the PVC filter was weighed three times using an electronic balance (Mettler-Toledo XP26, Resolution 0.001 mg)to calculate the average value (gravimetric analysis).

A PVC filter that collected RD following the gravimetric analysis was re-deposited in a silver filter in accordance with the NIOSH 7500 method, while X-ray diffraction (XRD) was used to analyze the RCS. For RCS, only quartz was detected, and the quartz concentration was quantified using the height of the peakrather than its area (to avoid interference from mullite and feldspar contained in the coal briquettes).The certified standard NISTSRM1878a was employed to obtain the calibrationcurve for the quartz concentration.The analysis took place at the Institute of Occupation and Environment, Korea Workers’ Compensation and Welfare Service, participating in the American Industrial Hygiene Association Proficiency Analytical Testing program. The analytical detection limit of quartz was 0.0025 mg/sample and the detection limit of the gravimetric analysis was 0.009 mg/sample.

Companies A and B collected RD using a disposable respirable PPI (parallel particle impactors, Cat No. 225-387), which collects air at a flow rate of 4 L/min, to increase the air sampling amount to improve the detection limit during silica analysis. Therefore, TD was not collected.

### Statistical analysis

In the RCS sample, 34 of the total 90 samples were not detected. When probability plots were drawn using the time-weighted average (TWA) values of RCS, RD, and TD, they were determined as right-skewed. When normality was examined at the 5% significance level by conducting Kolmogorov–Smirnov analysis, none of the three factors exhibited a normal distribution. After converting all data into natural log for statistical analysis, excluding non-detected samples, the Kolmogorov–Smirnov analysis results showed that two factors exhibited a lognormal distribution and the RCS samples showed linearity in a log probability plot. In addition, the RCS content (%) of RD was calculated and the following analysis was performed.

The geometric mean (GM), geometric standard deviation (GSD), and minimum and maximum values were calculated using descriptive statistics. All results were classified into environmental and occupational variables, such as the job task (collector, driver, street cleaner, blower, and CBA collector), season (spring, autumn, and winter), employment type (directly employed vs. employed by subcontractors), and city size (large vs. small cities). Table 2 shows the sample size of RCS by different variables. In the analysis of variance (ANOVA) analysis, the undetected RCS samples were converted to 0.001 mg/m^3^, which is the background concentration of quartz in the air. An ANOVA and Turkey’s HSD *post-hoc* test were conducted to examine whether there was a significant difference in TD, RD and RCS mean concentration and RCS content depending on the occupational and environmental variables. In addition Welch's tests were conducted when the homogeneity assumption of variance was violated.

**Table 2:** TD, RD and RCS sample size by different variables.

	Job task	N	Season	N	Employment type	N	City size	N
Varia-bles	Collector	42 (12)^a^	Spring	39 (9)	Directly employed	46 (16)	Large	43 (13)
	Driver	9 (0)						
			Autumn	35 (5)				
	Street cleaner	22 (1)						
					Employed by subcontractors	44 (14)	Small	47 (17)
	Blowing worker	13 (13)						
			Winter	16 (16)				
	CBA collector	4 (4)						
	Total	90 (30)		90 (30)		90 (30)		90 (30)

*TD* total dust, *RD* respirable dust, *RCS* respirable crystalline silica.

^a^Number of TD samples.

After converting all data into natural logs for Pearson correlation analysis, the results of the Shapiro–Wilk analysis showed that three factors, TD, RD, and RCS, exhibited a lognormal distribution. Pearson’s correlation analysis was conducted to examine the correlations among the RCS, RD, and TD exposure levels.

SPSS 23.0 software (IBM, Armonk, NY, USA) was used for statistical analysis, and figures were prepared using Origin 2016 software (OriginLab Co., Northampton, MA, USA).

### Ethical consideration

An ethical review and approval was not required for this study as this study did not involve humans or animals. This manuscript is only a study on the characteristics of dust in the air exposed to workers, and does not contain personal information, such as the age and gender of workers.

## Results

### RCS, RD, and TD exposure levels of sanitation workers

From various sanitation worker tasks, 90 RCS and RD samples together with 30 TD samples were collected. Table 3 shows the 8-h TWA concentrations of TD, RD, and RCS for each company. For RCS, 34 samples were less than the analytical detection limit. These samples were converted to 0.001 mg m^–3^, which is the value obtained by dividing the analytical detection limit of 0.0025 by 2.

**Table 3:** Exposure levels (mg m^–3^) of TD, RD, and RCS by company.

Companies	N	TD^a^		RD^b^		RCS^c^	
		GM^d^ (GSD^e^)	Range	GM (GSD)	Range	GM (GSD)	Range
A	30	–		0.082 (1.542)	0.052 ~ 0.240	0.001 (1.988)	0.001 ~ 0.007
B	30	–		0.033 (2.155)	0.004 ~ 0.115	0.003 (2.457)	0.001 ~ 0.015
C	5	0.042 (2.196)	0.014 ~ 0.106	0.016 (1.757)	0.007 ~ 0.027	0.002 (2.137)	0.001 ~ 0.004
D	2	7.385 (2.337)	4.052, 13.460	0.350 (1.649)	0.246, 0.499	0.019 (1.507)	0.014, 0.024
E	12	0.162 (1.727)	0.058 ~ 0.389	0.048 (1.578)	0.022 ~ 0.104	0.003 (1.235)	0.002 ~ 0.004
F	9	0.588 (1.454)	0.345 ~ 0.978	0.065 (1.392)	0.040 ~ 0.097	0.007 (1.462)	0.004 ~ 0.013
G	2	1.040 (1.122)	1.128, 0.958	0.126 (1.332)	0.103, 0.154	0.003 (1.417)	0.002, 0.004
Total	90	0.278 (4.168)	0.014 ~ 13.460	0.053 (2.205)	0.004 ~ 0.499	0.0026 (2.383)	0.001 ~ 0.024
Background level^f^		–	–	0.028 (1.545)	–	0.0007	–

^a^Total dust.

^b^Respirable dust.

^c^Respirable crystalline silica.

^d^Geometric mean.

^e^Geometric standard deviation.

^f^The background RCS and RD concentrations are the values obtained by the authors of this study through another research project in 2018^24^.

For the TD samples, the concentration ranged from 0.014 to 13.460 mg m^–3^, and the GM was 0.278 mg m^–3^. For the RD samples, the concentration ranged from 0.004 to 0.499 mg m^–3^, and the GM was 0.053 mg m^–3^. For the RCS samples, the concentration ranged from 0.001 to 0.024 mg m^–3^, and the GM was 0.0026 mg m^–3^, which is approximately four times higher than the background concentration in theatmosphere ^24^.

When the ANOVA was conducted, statistically significant differences were observed in RCS concentrations among the companies (p < 0.001).

### Relationships between RCS concentrations and various exposure factors

Table 4 shows the GM concentrations of TD, RD, and RCS according to the environmental and occupational factors of sanitation workers. CBA collectors exhibited the highest RCS GM concentration (0.0073 mg m^–3^), followed by workers who blow fallen leaves and sand using a blower (0.0051 mg m^–3^), collectors, street cleaners, and drivers (0.0026, 0.0017, and 0.0015 mg m^–3^) (p < 0.001). The GM concentration of the collectors was 2.6 µg m^–3^. However, 10 out of 21 samples exhibited values higher than 4 µg m^–3^, and the maximum concentration was 15 µg m^–3^, which was two-thirds the level of ACGIH TLV.

**Table 4:** TD, RD and RCS levels (µg/m^3^) according to occupational and working environment factors.

Variables		TD				RD				RCS			RCS/RD, %		
		N	GM (GSD)	P-value	Turkey’s HSD	N	GM (GSD)	P-value	Turkey’s HSD	GM (GSD)	P-value	Turkey’s HSD	GM	P-value	Turkey’s HSD
Job task	Collector^a^	12	0.162 (1.727)	0.010*	a,d < e^#^	42	0.050 (2.207)	**0.006**	a ~ d < e	0.0026 (2.194)	**0.000**	b,c < e	5	**0.004**	b,c,e < d
	Driver^b^	-	-			9	0.031 (2.081)			0.0015 (1.683)			3		
	Street cleaner^c^	1	0.106			22	0.046 (1.829)			0.0017 (2.294)			4		
	Blowing worker^d^	13	0.229 (4.290)			13	0.040 (2.259)			0.0051 (1.858)			12		
	CBA collector^e^	4	2.771 (3.440)			4	0.210 (1.972)			0.0073 (3.117)			6		
Season	Spring^a^	9	0.588 (1.454)	0.000*	b < a,c	39	0.065 (1.522)	0.000*	b < a,c	0.0021 (2.443)	0.019*	a < c	3	0.000*	a < b,c
	Autumn^b^	5	0.042 (2.196)			35	0.048 (2.176)			0.0026 (2.391)			4		
	Winter^c^	16	0.330 (4.308)			16	0.069 (2.286)			0.0040 (1.909)			6		
Employment type	Direct worker^a^	16	0.278 (4.124)	0.979	n.a	46	0.066 (1.938)	**0.002**	n.a	0.0025 (2.352)	**0.047**	n.a	4	0.000*	n.a
	Outsourcedworkers^b^	14	0.280 (4.450)			44	0.045 (2.336)			0.0031 (1.958)			5		
City scale	Small city^a^	13	0.406 (2.758)	**0.000**	n.a	43	0.053 (2.420)	0.091	n.a	0.0026 (2.541)	**0.000**	n.a	5	0.000*	n.a
	Big city^b^	17	0.125 (2.401)			47	0.058 (1.968)			0.0019 (1.997)			4		

Bold letters indicate that the difference is statistically significant.

*TD* total dust, *RD* respirable dust, *RCS* respirable crystalline silica, *CBA* used coal briquettes ash.

^#^Number of TSP sample in street cleaner is less than 2, so it is not included in the post-hoc analysis.

*Robust Tests of Equality of Means (Welch test).

**Figure 2:**
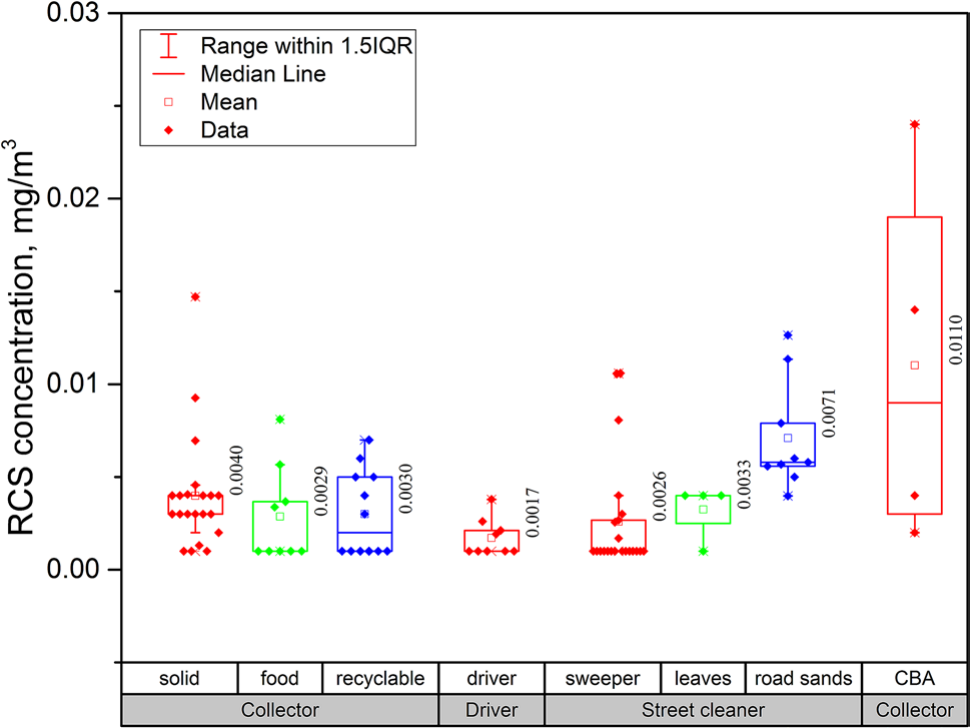
Box chart with data points overlap. Exposure level of respirable crystalline silica (RCS) by various tasks. Cleaning of fallen leaves and road sand uses a blower.

Figure 2 shows the RCS exposure levels of sanitation workers by work content in more detail than the results presented in Table 4. Collectors were subdivided into general, food, and recycling waste collectors. The blower task was divided into fallen leaves blowing and sand blowing (the mean concentration in Fig. 2 represents the arithmetic mean concentration). This indicates that the job task (work content) factor affected the levels of silica exposure experienced by sanitation workers.

The RCS GM concentration in winter was 0.0040 mg m^–3^, which was significantly higher than that in spring (0.0021 mg m^–3^) (p = 0.019). Workers employed by the subcontractors of local governments exhibited significantly greater exposure than those directly employed (0.0025 vs. 0.0031 mg m^–3^, p = 0.047). In the case of city size, small cities exhibited significantly higher exposure than larger cities (0.0019 vs. 0.0026 mg m^–3^, p < 0.001).

The RD GM concentrations of sanitation workers were affected by job task, season and employment type factors, but as for TD, the job task, season and city scale factor had a significant influence, while the employment type factor had no impact.

A more detailed description of ANOVA results by variable exposure factors is presented in the Supplementary information.

### Correlations between the TD, RD, and RCS concentration

The RCS concentration exhibited significant correlations with the RD and TD concentrations. The Pearson correlation coefficient between RCS and TD was 0.655 (p < 0.01), which was a high value and higher than between RCS and RD, which was 0.213 (p < 0.05), which was at a low level. This implies that when the RCS concentration increases, the concentration of TD, which represents large particles, also increases (Table 5).

**Table 5:** Pearson correlation coefficients among levels of RD, RCS and TD.

	RCS	RD	TD
RCS	1.000		
RD	0.213*	1.000	
TD	0.655**	0.877*	1.000

*Correlation is significant at the 0.05 level (two-tailed).

**Correlation is significant at the 0.01 level (two-tailed).

## Discussion

In South Korea, the incidence of lung cancer in sanitation workers is rising, which is leading to increased interest from governments and experts, thus, related studies have been conducted. Various improvement plans have also been discussed, including the application of improved waste transport vehicles, the introduction of early medical checkups for lung cancer, and the implementation of respiratory system protection programs.

Exposure to DPM is considered to be a major cause of lung cancer, but the DPM exposure level of sanitation workers is approximately GM 4.8 μg m^−3^ (1.7–29.0)^7^. Compared to the DPM level of underground mine workers which is approximately tens of micro grams per meter cube and sometimes exceeds 160  µg m^–3^ (the acceptable limit for mines in the United States)^25,26^, the DPM exposure level of sanitation workers found in this study is not significantly higher than that of other DPM exposure groups. Therefore, it is very important critical to recognize and investigate other carcinogens that may cause lung cancer in sanitation workers.

As the exposure of sanitation workers to RCS has previously not been recognized, this study may be the first to fully assess the RCS exposure level of sanitation workers.

The results of this study demonstrate the individual RCS exposure level of sanitation workers in seven companies and various job tasks. There were 34 non-detected samples out of a total of 90 samples. The arithmetic mean of 56 samples, excluding the non-detection samples, was 5.5 µg m^–3^ (GM 4.5), and after converting the non-detection samples into 1 µg m^–3^ using a conversion formula (LOD/2) the average concentration was 3.8 µg m^–3^ (GM 2.6). Considering that the background concentration of RCS in the atmosphere is 0.7 µg m^–3^ (this result was obtained by the authors through analyzing the quartz concentration after collecting RD in the atmosphere from Incheon City in 2018^24^), the RCS exposure level is approximately four times higher than the background concentration.

There is a lack of existing research on the silica exposure level of workers who work outdoors. Sanitation workers who work outdoors are also directly exposed to RCS in the atmosphere, and there are currently insufficient studies on the RCS concentration in the atmosphere. Thus, further studies are required to fully investigate this issue.

Of the limited research, a previous study showed that the RCS concentration of highway toll booth workers were below the detection limit^27^. Meanwhile, the GM RCS concentrations of workers who cultivated citrus and table grapes in farms in California, USA were as high as 0.08 and 0.02 mg m^–3^, respectively. This is likely because they were exposed to foliar dusts containing 10.1% and 7.9% quartz, respectively, resulting in very high TD concentrations of 41.8 and 3.5 mg m^–3^, respectively^28^. The RCS concentration in the atmosphere from an aggregate manufacturer in California, USA ranged from non-detection to 2.8 µg m^–3^, and the concentration exceeded 2.0 µg m^–3^ for all samples collected during the operation of the manufacturing facility^29^. In the UK, 80% of the samples collected from areas near seven construction sites and five sand quarries exhibited silica concentrations of less than 3.0 µg m^–3^, but the samples from the Sandstone Quarry exhibited concentrations of up to 21 µg m^−3^ downwind (the RCS concentration in rural background location was 0.02 µg m^–3^)^30^. In addition, similar to how sand dust generated from the Sahara Desert affects the surrounding areas and Europe^31^, Asian dust generated from China and Mongolia affects East Asian countries, including China, South Korea, and Japan. According to 2018 research conducted by the authors of this study, the RCS concentration was estimated to exceed the TLV with a value of 33 µg m^–3^ when a “yellow dust alert” is issued in South Korea as PM10 reaches 800 µg m^–3^^24^.

Therefore, when the RCS exposure of sanitation workers is assessed, it is necessary to consider the presence of aggregate companies, quarries, or yellow dust around the work area as the main variable.

In this study, the ANOVA analysis results confirmed that job task, season, employment type and city scale affect the RCS exposure of sanitation workers. Drivers exhibited the lowest RCS exposure level because they are usually in vehicles. Collectors and street cleaners demonstrated somewhat higher concentrations than drivers. This could be influenced by their exposure to the dust re-scattered from the ground while staying on the road for an extended period. According to Jancsek-Turóczi et al.^32^, dust re-scattered from roads in Veszprém, Hungary comprised of 10–20% quartz. In this study, when collectors were examined in detail, general waste collectors exhibited slightly higher silica concentrations than food and recycling waste collectors. This is likely because CBA with a high concentration of quartz is contained in general waste, and as such, its collectors are exposed to CBA dust while it is thrown into waste vehicles or while it is compressed.

In this study, two CBA powders obtained from two companies were scanned using XRD, and quartz (i.e. crystalline silica) was found in high concentrations. Tulepov et al.^33^ also confirmed that CBA contains quartz. Coal, the raw material of coal briquettes, contains less than 10% quartz^34^, however, carbon, the main component of coal, is pyrolyzed and volatilized when the coal briquettes are burned at 1000 °C or higher. Therefore, the quartz content per unit weight of the CBA is higher than that of the original coal briquettes. In addition, CBA is fragile and easily disintegrates to dust due to the low moisture content.

Although there are some differences depending on local governments, the task of the directly employed sanitation workers is to collect recycling waste, such as plastic waste and cans, rather than general waste which is likely to produce dust, such as CBA. In addition, as workers employed by subcontractors generally have a higher workload, it appears that the employment type also affects RCS concentration.

Among the RC workers, sweepers, who pick up roadside trash using tongs or perform sweeping, did not exhibit high RCS concentrations on average with many non-detection samples, but some samples exceeded the 10 µg m^–3^ level. This could be due to the existence of a large amount of roadside soil containing quartz that is swept in the work area. Meanwhile, the work of blowing fallen leaves and sand on the ground using a blower generated somewhat high RCS concentrations. In particular, the work of blowing sand involves the scattering a large amount of dust, resulting in a high TD concentration of approximately 1 mg m^–3^.

The RCS concentration was the highest for the CBA worker, but the RCS content of the RD was the highest, with an average of 12%, for the blower operator. This is because the concentration of RD, as well as RCS, is high in CBA work. It is well recognized that the blower work directly blows the sand off the road with a high content of quartz.

Every South Korean city disposes of CBA in different ways. In some areas, a large amount of CBA is discharged, and the largest task of sanitation workers is to collect and dispose the CBA. Although not included in this study, there were also some instances where CBA was thrown into an open cart and crushed by the feet in order to load more. In some apartments, CBA from each household was discharged through CBA discharge passages, and this CBA was then moved to a cart or a vehicle using shovels. In these work situations, sanitation workers can be expected to be exposed to higher levels than the RCS concentrations presented in this study. As CBA collectors are exposed to high TD and RCS concentrations, aggressive improvement measures are required. Moreover, as large amounts of CBA dust spread into the surroundings, in addition to affecting individual workers, the risk of exposure of local residents and environmental pollution also increases.

Some cities separately collect and reprocess CBA to use it as an industrial material, making it an exemplary mineral resource in terms of disposal cost reduction and recycling. This study, however, emphasizes the fact that a high concentration of carcinogenic quartz is contained in CBA, and that high exposure to RCS concentrations can occur during CBA handling. Therefore, the results of this study hold promising implications for industries and workers that reprocess CBA and could serve as basic data for health risk warnings.

Whereas, the results of the ANOVA test may not be robust, given that the sample sizes of different companies in this study vary significantly. Therefore, caution must be taken when drawing generalization on the variables affecting RCS exposure in this study. Further research may be needed with more diverse companies and work.

Although RCS was expected to have a high correlation with RD, it is quartz in RD that had a higher correlation with TD. While RD is significantly affected by various fine particles, including DPM, TD is affected by specific sources because it represents large particles. Crushed and scattered CBA dust and sand dust are scattered by blowing include RCS, which are essentially large particles. Therefore, when silica analysis is limited in terms of cost or time, it is advisable to evaluate TD as an alternative.

Although the RD and RCS exposure levels have shown a tendency to decrease since the global economic crisis of 2007–2008, traditional and occupational silica exposure groups are generally exposed to silica concentrations higher than tens of microgram per meter cube^35^. In the abovementioned well-known silica exposure groups, health management can be performed to protect the health of workers, such as by implementing respiratory system protection programs, applying engineering measures (e.g., dust collection using local exhaust devices in silica sources in a factory and the use of tools with dust suction devices during concrete drilling), and shift work. Sanitation workers who are not aware of silica exposure, however, have not been considered in health management strategies. It is also challenging to prepare improvement measures for RCS exposure reduction, considering the characteristics of their work.

However, many Asian countries use coal briquettes as fuel, and used CBA is disposed using different methods in each country and city. Regardless of the method, many sanitation workers are exposed to quartz contained in CBA dust, and sometimes even the general population in these areas can also be exposed.

As this study is the first dedicated research on RCS exposure of sanitation workers, further studies are required because it is estimated that sanitation workers with various tasks are exposed to silica in different countries and cities.

## Conclusions

This study is the first to measure the exposure level of RCS, a carcinogen, to sanitation workers who perform MHW collection or RC by work type. The goal was to investigate the RCS exposure characteristics of sanitation workers by identifying the individual RCS exposure levels of MHW and RC workers, as well as factors that may affect the exposure level.

The results indicate that job task, season, employment type and city scale are factors that impact the RCS exposure of sanitation workers. Although the overall average RCS exposure level was low, we have significant evidence to suggest that workers exposed to high concentrations of CBA dust and air blowing for road cleaning are also exposed to high concentrations of RCS.

## Supplementary Information

Supplementary Information.
