# Supplementary material for: Occupational exposure to respirable crystalline silica in municipal household waste collection and road cleaning workers
Source: Sci Rep. 2021 Jun 28;11:13370. doi: 10.1038/s41598-021-92809-5 (PMC8238943; doi:10.1038/s41598-021-92809-5)
Supplement: Supplementary file 1 — Supplementary Information. [file 41598_2021_92809_MOESM1_ESM.pdf]

Occupational Exposure to Respirable Crystalline Silica in Municipal Household Waste Collection and Road Cleaning Workers

Boowook Kim[1*], Eunyoung Kim[1], Wonseok Cha[1], Jungah Shin[1], Byung-Soon Choi[1], Daeho Kim[1], Miyeon Kim[1], Wonyang Kang[1], Sungwon Choi[1*]

[1]*Institute of Occupation and Environment, Korea Workers' Compensation and Welfare Service, 478, Munemi-ro, Incheon, Korea*.
*Corresponding author E-mail: labor7@gmail.com, c84one@kcomwel.or.kr

Supplemental document

Supplement 1. Results of Normality Test.
Supplement 2. Results of one-way ANOVA of TD by variable.
Supplement 3. Results of one-way ANOVA of RD by variable.
Supplement 4. Results of one-way ANOVA of RCS by variable.
Supplement 5. Results of one-way ANOVA of ratio of RCS in RD.

Supplement 1. Results of Normality test.

Investigating data of log-translated TD (total dust), RD (respirable dust), and RCS (respirable crystalline silica) concentrations was performed using SPSS software. TD and RD showed Normality in Kolmogorov-Smirnov and Shapiro-Wilk analysis results, and RCS showed a lognormal distribution when observed histograms and Q-Q plots.

Explore

Case Processing Summary

	Cases					
	Valid		Missing		Total	
	N	Percent	N	Percent	N	Percent
Ln_TD	30	100.0%	0	0.0%	30	100.0%

Descriptives

			Statistic	Std. Error
Ln_TD	Mean		-1.2796	.26062
	95% Confidence Interval for Mean	Lower Bound	-1.8126	
		Upper Bound	-.7466	
	5% Trimmed Mean		-1.3227	
	Median		-1.3724	
	Variance		2.038	
	Std. Deviation		1.42748	
	Minimum		-4.27	
	Maximum		2.60	
	Range		6.87	
	Interquartile Range		1.85	
	Skewness		.409	.427
	Kurtosis		.996	.833

Tests of Normality

	Kolmogorov-Smirnov[a]			Shapiro-Wilk		
	Statistic	df	Sig.	Statistic	df	Sig.
Ln_TD	.097	30	.200*	.978	30	.757

*. This is a lower bound of the true significance.

a. Lilliefors Significance Correction

Ln_TD

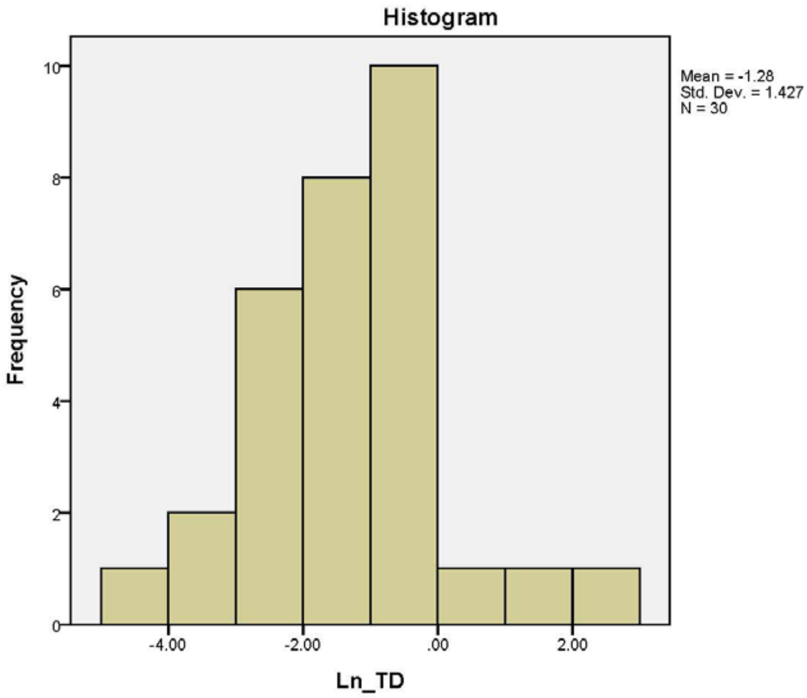

Ln_TD Stem-and-Leaf Plot

Frequency	Stem &	Leaf
1.00	-4 .	2
2.00	-3 .	34
6.00	-2 .	024468
8.00	-1 .	03345689
10.00	-0 .	0011556699
1.00	0 .	1
1.00	1 .	3
1.00 Extremes		(>=2.6)

Stem width: 1.00
Each leaf: 1 case(s)

쪽 2

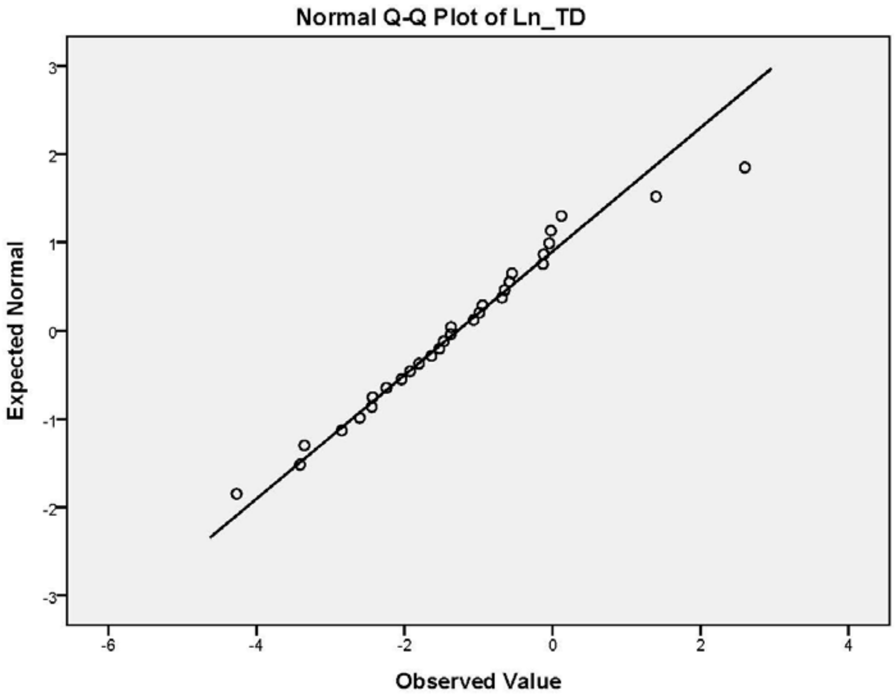
Normal Q-Q Plot of Ln_TD
Expected Normal
Observed Value
3
2
1
0
-1
-2
-3
-6
-4
-2
0
2
4

쪽 3

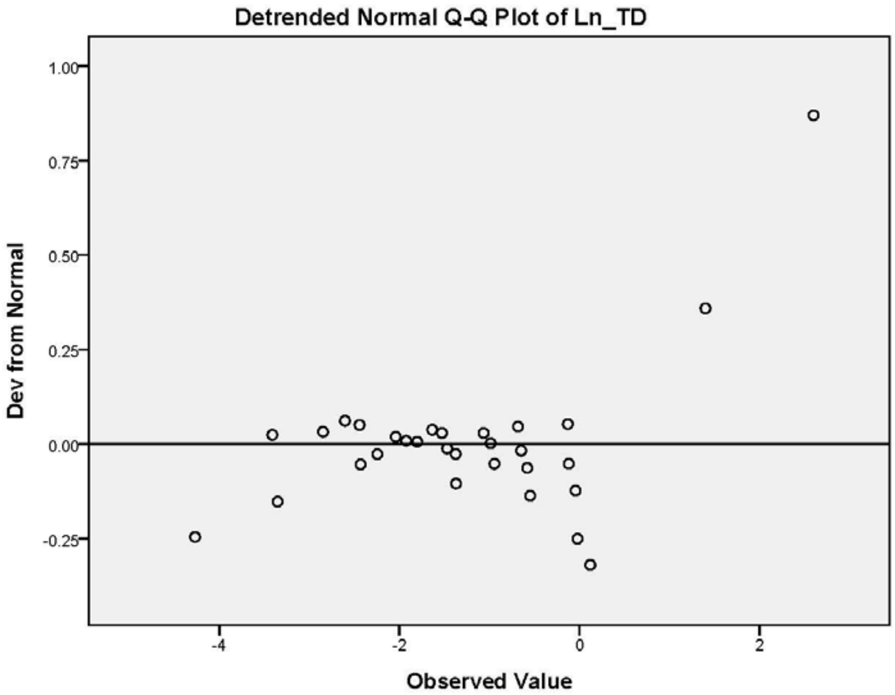

Case Processing Summary

	Cases					
	Valid		Missing		Total	
	N	Percent	N	Percent	N	Percent
Ln_RD	56	100.0%	0	0.0%	56	100.0%
Ln_RCS	56	100.0%	0	0.0%	56	100.0%

Descriptives

			Statistic	Std. Error
Ln_RD	Mean		-2.9346	.10812
	95% Confidence Interval for Mean	Lower Bound	-3.1513	
		Upper Bound	-2.7180	
	5% Trimmed Mean		-2.9277	
	Median		-2.8473	
	Variance		.655	
	Std. Deviation		.80911	
	Minimum		-4.96	
	Maximum		-.70	
	Range		4.27	
	Interquartile Range		.90	
	Skewness		-.190	.319
	Kurtosis		.951	.628
Ln_RCS	Mean		-5.3857	.07869
	95% Confidence Interval for Mean	Lower Bound	-5.5434	
		Upper Bound	-5.2280	
	5% Trimmed Mean		-5.4001	
	Median		-5.5215	
	Variance		.347	
	Std. Deviation		.58885	
	Minimum		-6.91	
	Maximum		-3.73	
	Range		3.18	
	Interquartile Range		.69	
	Skewness		.434	.319
	Kurtosis		.630	.628

Tests of Normality

	Kolmogorov-Smirnov[a]			Shapiro-Wilk		
	Statistic	df	Sig.	Statistic	df	Sig.
Ln_RD	.096	56	.200*	.973	56	.239
Ln_RCS	.180	56	.000	.958	56	.047

*. This is a lower bound of the true significance.

a. Lilliefors Significance Correction

Ln_RD

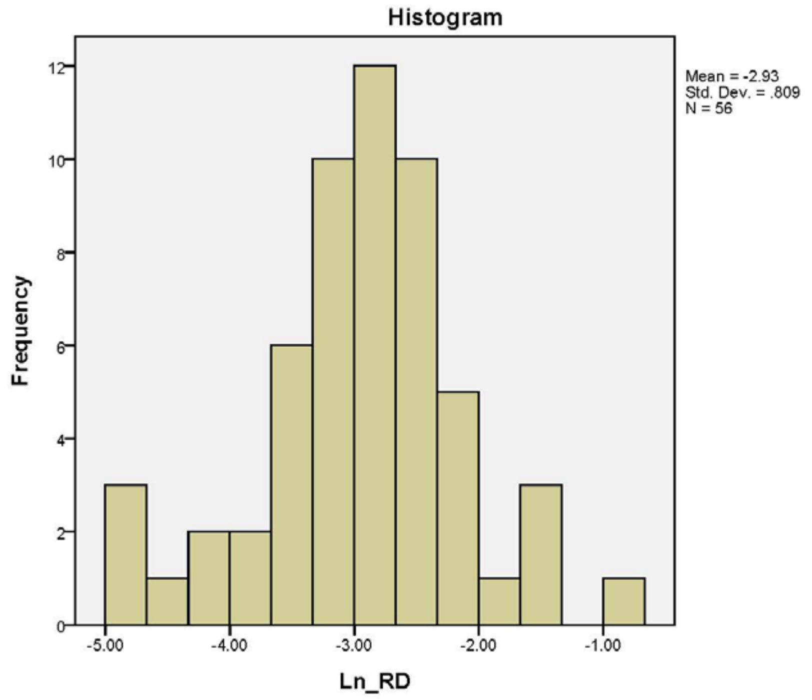


```
Ln_RD Stem-and-Leaf Plot

 Frequency    Stem &  Leaf

     3.00 Extremes    (=<-4.7)
     3.00       -4 .  014
     5.00       -3 .  66668
    13.00       -3 .  0000112223334
    17.00       -2 .  55556667777788999
    10.00       -2 .  1222333444
     3.00       -1 .  568
     1.00       -1 .  4
     1.00 Extremes    (>=-.7)

 Stem width:      1.00
 Each leaf:        1 case(s)
```

쪽 6

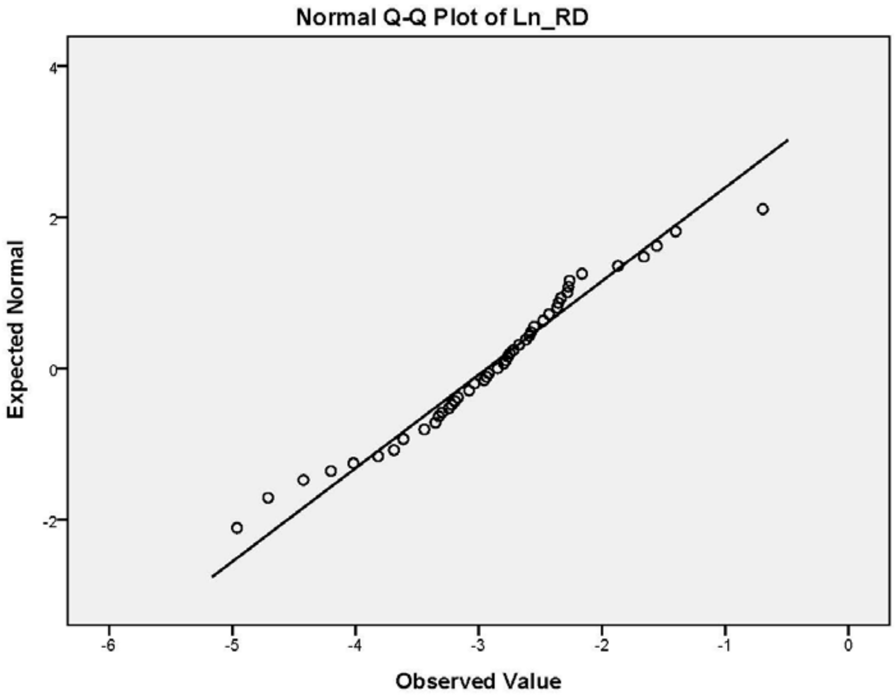
Normal Q-Q Plot of Ln_RD
Expected Normal
Observed Value
4
2
0
-2
-6
-5
-4
-3
-2
-1
0

쪽 7

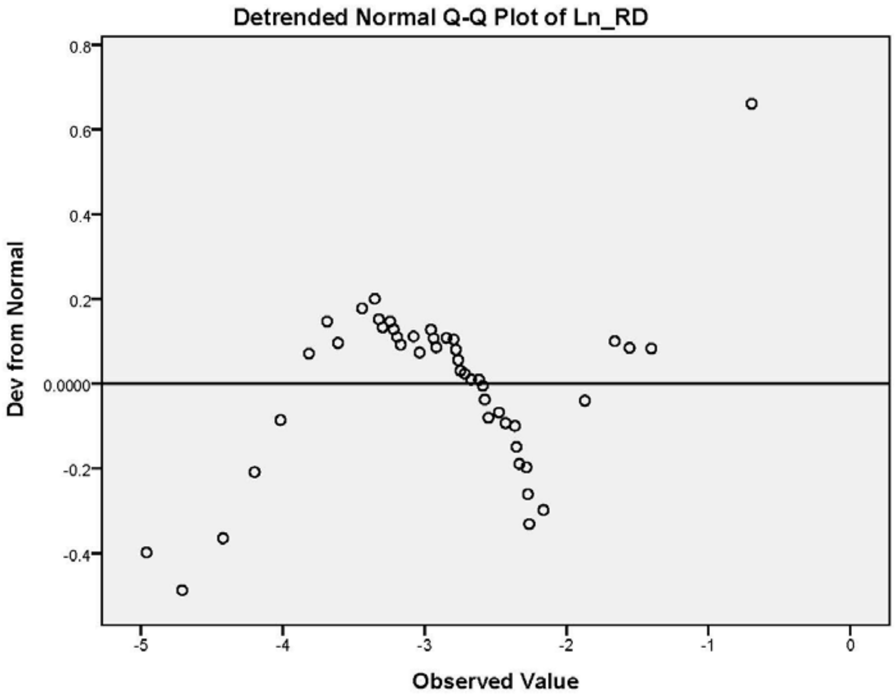
Detrended Normal Q-Q Plot of Ln_RD
Dev from Normal
Observed Value
0.8
0.6
0.4
0.2
0.0000
-0.2
-0.4
-5
-4
-3
-2
-1
0

Ln_RCS

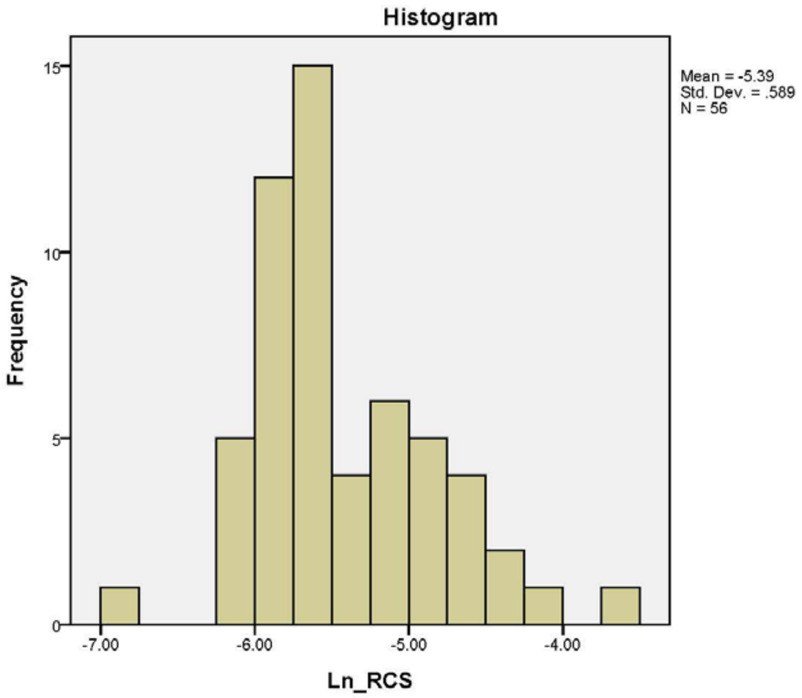


```
Ln_RCS Stem-and-Leaf Plot

 Frequency    Stem &  Leaf

     1.00 Extremes    (=<-6.9)
     5.00       -6 .  12222
    27.00       -5 .  555555555555556888888888888
    10.00       -5 .  1111112222
     9.00       -4 .  555788899
     3.00       -4 .  123
     1.00 Extremes    (>=-3.7)

 Stem width:      1.00
 Each leaf:        1 case(s)
```

쪽 9

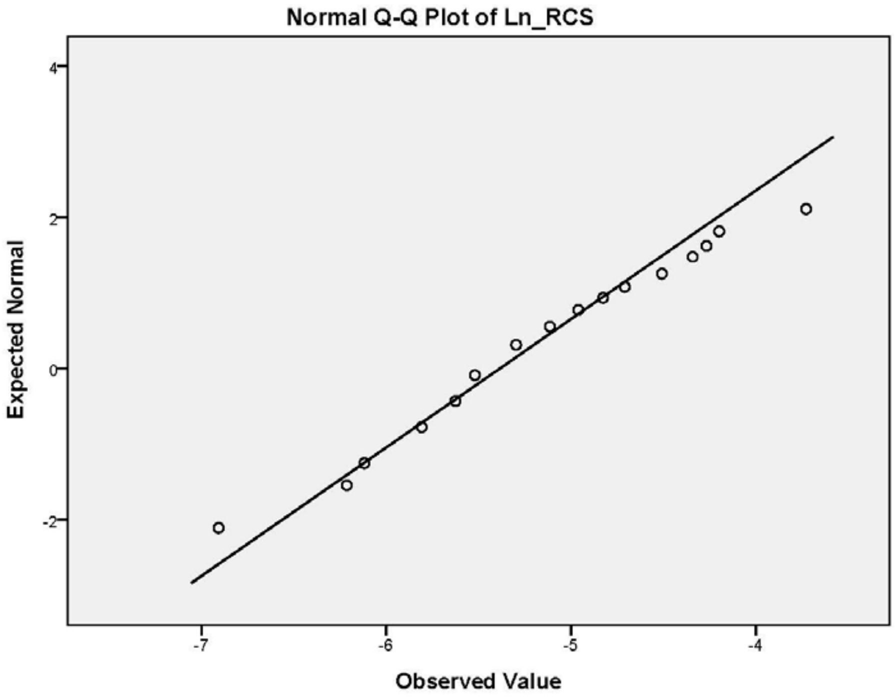
Normal Q-Q Plot of Ln_RCS
Expected Normal
Observed Value
4
2
0
-2
-7
-6
-5
-4

쪽 10

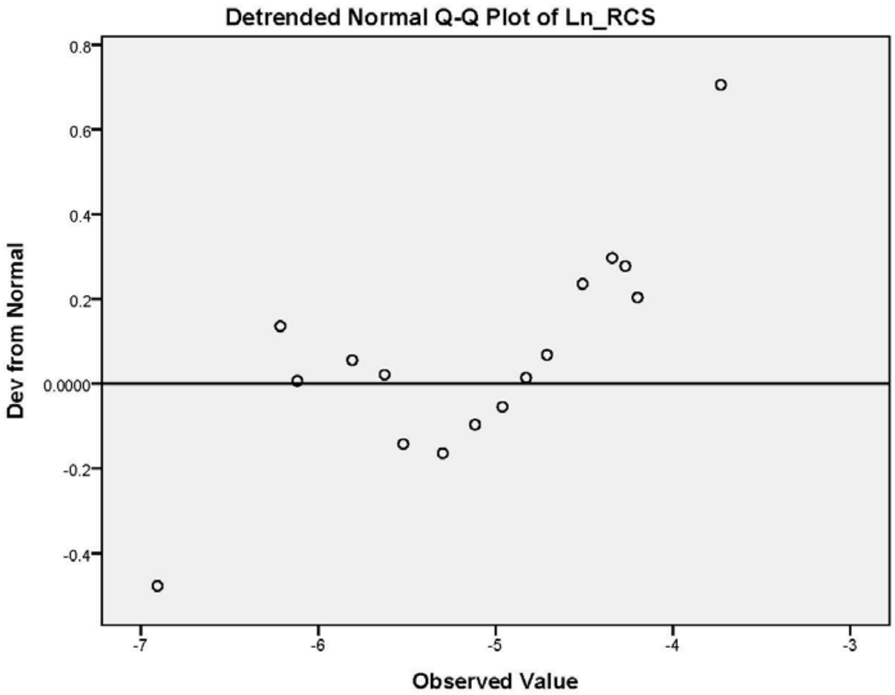
Detrended Normal Q-Q Plot of Ln_RCS
Dev from Normal
0.8
0.6
0.4
0.2
0.0000
-0.2
-0.4
-7
-6
-5
-4
-3
Observed Value

쪽 11

Supplement 2. Results of one-way ANOVA of TD by variable.

Supplement 2 presents the results of a one-way ANOVA of TD concentration by variable. In the job variables, the variance of each group was shown to be different, with Levine's statistics of less than 0.05, and Brown-Forsythe test and Welch test were performed. The result was statistically significant with a significance probability of 0.01 in Welch test.
As a result of the Turkey'sHSD *post-hoc*analysis, TD concentration of CBA collectors were significantly higher than that of general collectors and air blowing workers.
In the season variables, the variance of each group was shown to be different, with Levine's statistics below 0.05, and the results were statistically significant below 0.01.
Turkey's *post-hoc* analysis found that TD concentrations in spring and winter were significantly higher than in fall.
By city size, TD concentrations in small cities were statistically significantly higher than in large cities. However, there was no difference in TD concentration by employment type.

```
ONEWAY Ln_TSP BY Job_5
  /STATISTICS HOMOGENEITY WELCH
  /MISSING ANALYSIS
  /POSTHOC=TUKEY ALPHA(0.05).
```

Oneway

Test of Homogeneity of Variances

Ln_TSP

Levene Statistic	df1	df2	Sig.
6.758	2	26	.004

ANOVA

Ln_TSP

	Sum of Squares	df	Mean Square	F	Sig.
Between Groups	24.817	2	12.408	9.684	.001
Within Groups	33.314	26	1.281		
Total	58.131	28			

Robust Tests of Equality of Means

Ln_TSP

	Statistic[a]	df1	df2	Sig.
Welch	9.196	2	7.301	.010

a. Asymptotically F distributed.

Post Hoc Tests

쪽 1

Multiple Comparisons

Dependent Variable: Ln_TSP

Tukey HSD

(I) Job_5	(J) Job_5	Mean Difference (I-J)	Std. Error	Sig.	95% Confidence Interval	
					Lower Bound	Upper Bound
Collector	Blower	-.40082	.45314	.655	-1.5268	.7252
	CBA.collector	-2.83637*	.65353	.001	-4.4603	-1.2124
Blower	Collector	.40082	.45314	.655	-.7252	1.5268
	CBA.collector	-2.43555*	.64722	.002	-4.0438	-.8273
CBA.collector	Collector	2.83637*	.65353	.001	1.2124	4.4603
	Blower	2.43555*	.64722	.002	.8273	4.0438

*. The mean difference is significant at the 0.05 level.

Homogeneous Subsets

Ln_TSP

Tukey HSD[a,b]

Job_5	N	Subset for alpha = 0.05	
		1	2
Collector	12	-1.8173	
Blower	13	-1.4164	
CBA.collector	4		1.0191
Sig.		.779	1.000

Means for groups in homogeneous subsets are displayed.

a. Uses Harmonic Mean Sample Size = 7.313.

b. The group sizes are unequal. The harmonic mean of the group sizes is used. Type I error levels are not guaranteed.

```
ONEWAY Ln_TSP BY Season
  /STATISTICS HOMOGENEITY WELCH
  /MISSING ANALYSIS
  /POSTHOC=TUKEY ALPHA(0.05).
```

Oneway

Test of Homogeneity of Variances

Ln_TSP

Levene Statistic	df1	df2	Sig.
3.575	2	27	.042

ANOVA

Ln_TSP

	Sum of Squares	df	Mean Square	F	Sig.
Between Groups	23.499	2	11.750	8.913	.001
Within Groups	35.594	27	1.318		
Total	59.094	29			

Robust Tests of Equality of Means

Ln_TSP

	Statistic[a]	df1	df2	Sig.
Welch	23.817	2	10.200	.000

a. Asymptotically F distributed.

Post Hoc Tests

Multiple Comparisons

Dependent Variable: Ln_TSP

Tukey HSD

(I) Season	(J) Season	Mean Difference (I-J)	Std. Error	Sig.	95% Confidence Interval Lower Bound	95% Confidence Interval Upper Bound
Spring	Autumn	2.64523*	.64042	.001	1.0574	4.2331
	winter	.57732	.47841	.460	-.6089	1.7635
Autumn	Spring	-2.64523*	.64042	.001	-4.2331	-1.0574
	winter	-2.06791*	.58827	.004	-3.5265	-.6094
winter	Spring	-.57732	.47841	.460	-1.7635	.6089
	Autumn	2.06791*	.58827	.004	.6094	3.5265

*. The mean difference is significant at the 0.05 level.

Homogeneous Subsets

쪽 3

Ln_TSP

Tukey HSD[a,b]

Season	N	Subset for alpha = 0.05	
		1	2
Autumn	5	-3.1761	
winter	16		-1.1082
Spring	9		-.5308
Sig.		1.000	.579

Means for groups in homogeneous subsets are displayed.

a. Uses Harmonic Mean Sample Size = 8.030.

b. The group sizes are unequal. The harmonic mean of the group sizes is used. Type I error levels are not guaranteed.

```
ONEWAY Ln_TSP BY Employment
  /STATISTICS HOMOGENEITY WELCH
  /MISSING ANALYSIS
  /POSTHOC=TUKEY ALPHA(0.05).
```

Oneway

Warnings

Post hoc tests are not performed for Ln_TSP because there are fewer than three groups.

Test of Homogeneity of Variances

Ln_TSP

Levene Statistic	df1	df2	Sig.
.358	1	28	.555

ANOVA

Ln_TSP

	Sum of Squares	df	Mean Square	F	Sig.
Between Groups	.002	1	.002	.001	.979
Within Groups	59.092	28	2.110		
Total	59.094	29			

Robust Tests of Equality of Means

Ln_TSP

	Statistic[a]	df1	df2	Sig.
Welch	.001	1	27.020	.979

a. Asymptotically F distributed.

```
ONEWAY Ln_TSP BY City_2
  /STATISTICS HOMOGENEITY WELCH
  /MISSING ANALYSIS
  /POSTHOC=TUKEY ALPHA(0.05).
```

Oneway

Warnings

Post hoc tests are not performed for Ln_TSP because there are fewer than three groups.

Test of Homogeneity of Variances

Ln_TSP

Levene Statistic	df1	df2	Sig.
.012	1	28	.915

ANOVA

Ln_TSP

	Sum of Squares	df	Mean Square	F	Sig.
Between Groups	34.465	1	34.465	39.182	.000
Within Groups	24.629	28	.880		
Total	59.094	29			

Robust Tests of Equality of Means

Ln_TSP

	Statistic[a]	df1	df2	Sig.
Welch	37.634	1	23.784	.000

a. Asymptotically F distributed.

Supplement 3. Results of one-way ANOVA of RD by variable.

Supplement3 presents the results of a one-way ANOVA of RD concentration by variable.

RD concentrations showed statistically significant differences depending on job, season and employment type. However, RD concentrations with city size showed no significant differences.
In the job variables, as a result of the Turkey's HSD *post-hoc*analysis, RD concentration of CBA collectors were significantly higher than that of general collectors and air blowing workers.
In the season variables, Turkey's *post-hoc* analysis found that RD concentrations in spring and winter were significantly higher than in fall.
By employment type, RD concentrations in direct workers were statistically significantly higher than in outsourced workers. However, there was no difference in TD concentration by city scale.

```
ONEWAY Ln_RSP BY Job_5
  /STATISTICS HOMOGENEITY WELCH
  /MISSING ANALYSIS
  /POSTHOC=TUKEY ALPHA(0.05).
```

Oneway

Test of Homogeneity of Variances

Ln_RSP

Levene Statistic	df1	df2	Sig.
.261	4	85	.902

ANOVA

Ln_RSP

	Sum of Squares	df	Mean Square	F	Sig.
Between Groups	8.664	4	2.166	3.916	.006
Within Groups	47.014	85	.553		
Total	55.678	89			

Robust Tests of Equality of Means

Ln_RSP

	Statistic[a]	df1	df2	Sig.
Welch	4.073	4	16.090	.018

a. Asymptotically F distributed.

Post Hoc Tests

Multiple Comparisons

Dependent Variable: Ln_RSP

Tukey HSD

(I) Job_5	(J) Job_5	Mean Difference (I-J)	Std. Error	Sig.	95% Confidence Interval Lower Bound	95% Confidence Interval Upper Bound
Collector	Driver	.11682	.27318	.993	-.6446	.8782
	St.sweeper	.08647	.19573	.992	-.4591	.6320
	Blower	.24873	.23604	.829	-.4092	.9066
	CBA.collector	-1.37637*	.38916	.006	-2.4610	-.2917
Driver	Collector	-.11682	.27318	.993	-.8782	.6446
	St.sweeper	-.03035	.29427	1.000	-.8505	.7899
	Blower	.13192	.32249	.994	-.7669	1.0308
	CBA.collector	-1.49319*	.44691	.011	-2.7388	-.2476
St.sweeper	Collector	-.08647	.19573	.992	-.6320	.4591
	Driver	.03035	.29427	1.000	-.7899	.8505
	Blower	.16226	.26017	.971	-.5629	.8874
	CBA.collector	-1.46284*	.40425	.004	-2.5896	-.3361
Blower	Collector	-.24873	.23604	.829	-.9066	.4092
	Driver	-.13192	.32249	.994	-1.0308	.7669
	St.sweeper	-.16226	.26017	.971	-.8874	.5629
	CBA.collector	-1.62510*	.42523	.002	-2.8103	-.4399
CBA.collector	Collector	1.37637*	.38916	.006	.2917	2.4610
	Driver	1.49319*	.44691	.011	.2476	2.7388
	St.sweeper	1.46284*	.40425	.004	.3361	2.5896
	Blower	1.62510*	.42523	.002	.4399	2.8103

*. The mean difference is significant at the 0.05 level.

Homogeneous Subsets

Ln_RSP

Tukey HSD[a,b]

Job_5	N	Subset for alpha = 0.05 1	2
Blower	13	-3.1866	
Driver	9	-3.0547	
St.sweeper	22	-3.0243	
Collector	42	-2.9379	
CBA.collector	4		-1.5615
Sig.		.946	1.000

Means for groups in homogeneous subsets are displayed.

a. Uses Harmonic Mean Sample Size = 9.856.

b. The group sizes are unequal. The harmonic mean of the group sizes is used. Type I error levels are not guaranteed.

```
ONEWAY Ln_RSP BY Season
  /STATISTICS HOMOGENEITY WELCH
  /MISSING ANALYSIS
  /POSTHOC=TUKEY ALPHA(0.05).
```

Oneway

Test of Homogeneity of Variances

Ln_RSP

Levene Statistic	df1	df2	Sig.
5.338	2	87	.007

ANOVA

Ln_RSP

	Sum of Squares	df	Mean Square	F	Sig.
Between Groups	18.160	2	9.080	21.055	.000
Within Groups	37.518	87	.431		
Total	55.678	89			

쪽 3

Robust Tests of Equality of Means

Ln_RSP

	Statistic[a]	df1	df2	Sig.
Welch	20.452	2	34.237	.000

a. Asymptotically F distributed.

Post Hoc Tests

Multiple Comparisons

Dependent Variable: Ln_RSP

Tukey HSD

(I) Season	(J) Season	Mean Difference (I-J)	Std. Error	Sig.	95% Confidence Interval Lower Bound	Upper Bound
Spring	Autumn	.95190*	.15290	.000	.5873	1.3165
	winter	.11876	.19496	.816	-.3461	.5836
Autumn	Spring	-.95190*	.15290	.000	-1.3165	-.5873
	winter	-.83314*	.19818	.000	-1.3057	-.3606
winter	Spring	-.11876	.19496	.816	-.5836	.3461
	Autumn	.83314*	.19818	.000	.3606	1.3057

*. The mean difference is significant at the 0.05 level.

Homogeneous Subsets

Ln_RSP

Tukey HSD[a,b]

Season	N	Subset for alpha = 0.05: 1	2
Autumn	35	-3.5060	
winter	16		-2.6729
Spring	39		-2.5541
Sig.		1.000	.794

Means for groups in homogeneous subsets are displayed.

a. Uses Harmonic Mean Sample Size = 25.704.

b. The group sizes are unequal. The harmonic mean of the group sizes is used. Type I error levels are not guaranteed.

```
ONEWAY Ln_RSP BY Employment
  /STATISTICS HOMOGENEITY WELCH
  /MISSING ANALYSIS
  /POSTHOC=TUKEY ALPHA(0.05).
```

Oneway

Warnings

Post hoc tests are not performed for Ln_RSP because there are fewer than three groups.

Test of Homogeneity of Variances

Ln_RSP

Levene Statistic	df1	df2	Sig.
2.015	1	88	.159

ANOVA

Ln_RSP

	Sum of Squares	df	Mean Square	F	Sig.
Between Groups	5.523	1	5.523	9.690	.002
Within Groups	50.155	88	.570		
Total	55.678	89			

Robust Tests of Equality of Means

Ln_RSP

	Statistic[a]	df1	df2	Sig.
Welch	9.588	1	81.625	.003

a. Asymptotically F distributed.

```
ONEWAY Ln_RSP BY City_2
  /STATISTICS HOMOGENEITY WELCH
  /MISSING ANALYSIS
  /POSTHOC=TUKEY ALPHA(0.05).
```

Oneway

Warnings

Post hoc tests are not performed for Ln_RSP because there are fewer than three groups.

Test of Homogeneity of Variances

Ln_RSP

Levene Statistic	df1	df2	Sig.
2.535	1	88	.115

ANOVA

Ln_RSP

	Sum of Squares	df	Mean Square	F	Sig.
Between Groups	1.785	1	1.785	2.914	.091
Within Groups	53.893	88	.612		
Total	55.678	89			

Robust Tests of Equality of Means

Ln_RSP

	Statistic[a]	df1	df2	Sig.
Welch	2.847	1	78.526	.096

a. Asymptotically F distributed.

Supplement 4. Results of one-way ANOVA of RCS by variable.

Supplement4 presents the results of a one-way ANOVA of RCS concentration by variable. RCS concentrations showed statistically significant differences depending on job, season, employment type, and city scale. As with TD and RD results, RCS concentrations in CBA collectors, winter, and small cities were the highest. However, unlike RD, the RCS concentration of directly employed workers was higher than that of outsourced workers.

```
ONEWAY Ln_RCS BY Job_5
  /STATISTICS HOMOGENEITY WELCH
  /MISSING ANALYSIS
  /POSTHOC=TUKEY ALPHA(0.05).
```

Oneway

Test of Homogeneity of Variances

Ln_RCS

Levene Statistic	df1	df2	Sig.
2.374	4	85	.058

ANOVA

Ln_RCS

	Sum of Squares	df	Mean Square	F	Sig.
Between Groups	16.257	4	4.064	6.666	.000
Within Groups	51.821	85	.610		
Total	68.078	89			

Robust Tests of Equality of Means

Ln_RCS

	Statistic[a]	df1	df2	Sig.
Welch	7.154	4	16.099	.002

a. Asymptotically F distributed.

Post Hoc Tests

쪽 1

Multiple Comparisons

Dependent Variable: Ln_RCS

Tukey HSD

(I) Job_5	(J) Job_5	Mean Difference (I-J)	Std. Error	Sig.	95% Confidence Interval Lower Bound	Upper Bound
Collector	Driver	.51893	.28680	.375	-.2804	1.3183
	St.sweeper	.39222	.20549	.321	-.1805	.9650
	Blower	-.69431*	.24782	.048	-1.3850	-.0036
	CBA.collector	-1.02256	.40857	.100	-2.1613	.1162
Driver	Collector	-.51893	.28680	.375	-1.3183	.2804
	St.sweeper	-.12671	.30895	.994	-.9878	.7344
	Blower	-1.21325*	.33858	.005	-2.1569	-.2696
	CBA.collector	-1.54149*	.46921	.013	-2.8493	-.2337
St.sweeper	Collector	-.39222	.20549	.321	-.9650	.1805
	Driver	.12671	.30895	.994	-.7344	.9878
	Blower	-1.08654*	.27315	.001	-1.8479	-.3252
	CBA.collector	-1.41478*	.42441	.011	-2.5977	-.2319
Blower	Collector	.69431*	.24782	.048	.0036	1.3850
	Driver	1.21325*	.33858	.005	.2696	2.1569
	St.sweeper	1.08654*	.27315	.001	.3252	1.8479
	CBA.collector	-.32825	.44644	.948	-1.5726	.9161
CBA.collector	Collector	1.02256	.40857	.100	-.1162	2.1613
	Driver	1.54149*	.46921	.013	.2337	2.8493
	St.sweeper	1.41478*	.42441	.011	.2319	2.5977
	Blower	.32825	.44644	.948	-.9161	1.5726

*. The mean difference is significant at the 0.05 level.

Homogeneous Subsets

Ln_RCS

Tukey HSD[a,b]

Job_5	N	Subset for alpha = 0.05		
		1	2	3
Driver	9	-6.4776		
St.sweeper	22	-6.3509		
Collector	42	-5.9587	-5.9587	
Blower	13		-5.2644	-5.2644
CBA.collector	4			-4.9361
Sig.		.581	.288	.883

Means for groups in homogeneous subsets are displayed.

a. Uses Harmonic Mean Sample Size = 9.856.

b. The group sizes are unequal. The harmonic mean of the group sizes is used. Type I error levels are not guaranteed.

```
ONEWAY Ln_RCS BY Season
  /STATISTICS HOMOGENEITY WELCH
  /MISSING ANALYSIS
  /POSTHOC=TUKEY ALPHA(0.05).
```

Oneway

Test of Homogeneity of Variances

Ln_RCS

Levene Statistic	df1	df2	Sig.
8.412	2	87	.000

ANOVA

Ln_RCS

	Sum of Squares	df	Mean Square	F	Sig.
Between Groups	4.601	2	2.301	3.153	.048
Within Groups	63.477	87	.730		
Total	68.078	89			

Robust Tests of Equality of Means

Ln_RCS

	Statistic[a]	df1	df2	Sig.
Welch	4.346	2	47.220	.019

a. Asymptotically F distributed.

Post Hoc Tests

Multiple Comparisons

Dependent Variable: Ln_RCS

Tukey HSD

(I) Season	(J) Season	Mean Difference (I-J)	Std. Error	Sig.	95% Confidence Interval Lower Bound	95% Confidence Interval Upper Bound
Spring	Autumn	-.22443	.19888	.499	-.6987	.2498
	winter	-.63447*	.25359	.037	-1.2392	-.0298
Autumn	Spring	.22443	.19888	.499	-.2498	.6987
	winter	-.41003	.25777	.255	-1.0247	.2046
winter	Spring	.63447*	.25359	.037	.0298	1.2392
	Autumn	.41003	.25777	.255	-.2046	1.0247

*. The mean difference is significant at the 0.05 level.

Homogeneous Subsets

Ln_RCS

Tukey HSD[a,b]

Season	N	Subset for alpha = 0.05 1	Subset for alpha = 0.05 2
Spring	39	-6.1608	
Autumn	35	-5.9364	-5.9364
winter	16		-5.5263
Sig.		.615	.203

Means for groups in homogeneous subsets are displayed.

a. Uses Harmonic Mean Sample Size = 25.704.

b. The group sizes are unequal. The harmonic mean of the group sizes is used. Type I error levels are not guaranteed.

쪽 4

```
ONEWAY Ln_RCS BY Employment
  /STATISTICS HOMOGENEITY WELCH
  /MISSING ANALYSIS
  /POSTHOC=TUKEY ALPHA(0.05).
```

Oneway

Warnings

Post hoc tests are not performed for Ln_RCS because there are fewer than three groups.

Test of Homogeneity of Variances

Ln_RCS

Levene Statistic	df1	df2	Sig.
2.380	1	88	.126

ANOVA

Ln_RCS

	Sum of Squares	df	Mean Square	F	Sig.
Between Groups	2.993	1	2.993	4.046	.047
Within Groups	65.086	88	.740		
Total	68.078	89			

Robust Tests of Equality of Means

Ln_RCS

	Statistic[a]	df1	df2	Sig.
Welch	4.046	1	87.824	.047

a. Asymptotically F distributed.

```
ONEWAY Ln_RCS BY City_2
  /STATISTICS HOMOGENEITY WELCH
  /MISSING ANALYSIS
  /POSTHOC=TUKEY ALPHA(0.05).
```

Oneway

Warnings

Post hoc tests are not performed for Ln_RCS because there are fewer than three groups.

쪽 5

Test of Homogeneity of Variances

Ln_RCS

Levene Statistic	df1	df2	Sig.
2.950	1	88	.089

ANOVA

Ln_RCS

	Sum of Squares	df	Mean Square	F	Sig.
Between Groups	8.991	1	8.991	13.390	.000
Within Groups	59.087	88	.671		
Total	68.078	89			

Robust Tests of Equality of Means

Ln_RCS

	Statistic[a]	df1	df2	Sig.
Welch	13.037	1	76.727	.001

a. Asymptotically F distributed.

쪽 6

Supplement 5. Results of one-way ANOVA of ratio of RCS in RD.

Supplement5 presents the results of a one-way ANOVA of RCS ratios among RD by variable. RCS ratios, like RCS concentrations, showed statistically significant differences depending on job, season, employment type and city size. However, the RCS concentration was the highest in CBA collection workers, while the RCS ratios were the highest in airblower workers. This is because Company F removed the sand (very high quartz content) sprayed on the road.

```
EXAMINE VARIABLES=Ln_ratio
  /PLOT BOXPLOT STEMLEAF NPPLOT
  /COMPARE GROUPS
  /STATISTICS DESCRIPTIVES
  /CINTERVAL 95
  /MISSING LISTWISE
  /NOTOTAL.
```

Explore

Case Processing Summary

	Cases					
	Valid		Missing		Total	
	N	Percent	N	Percent	N	Percent
Ln_ratio	90	100.0%	0	0.0%	90	100.0%

Descriptives

			Statistic	Std. Error
Ln_ratio	Mean		-3.0222	.10951
	95% Confidence Interval for Mean	Lower Bound	-3.2398	
		Upper Bound	-2.8046	
	5% Trimmed Mean		-3.0359	
	Median		-2.8645	
	Variance		1.079	
	Std. Deviation		1.03886	
	Minimum		-5.48	
	Maximum		-.05	
	Range		5.43	
	Interquartile Range		1.67	
	Skewness		.091	.254
	Kurtosis		-.039	.503

Tests of Normality

	Kolmogorov-Smirnov[a]			Shapiro-Wilk		
	Statistic	df	Sig.	Statistic	df	Sig.
Ln_ratio	.081	90	.196	.984	90	.325

a. Lilliefors Significance Correction

Ln_ratio

쪽 1

```
Ln_ratio Stem-and-Leaf Plot

 Frequency    Stem &  Leaf

     2.00       -5 .  34
    18.00       -4 .  000111112223334458
    21.00       -3 .  001223333455567889999
    36.00       -2 .  000002222233344445566667777778888999
    11.00       -1 .  02257889999
     2.00       -0 .  05

 Stem width:      1.00
 Each leaf:        1 case(s)
```

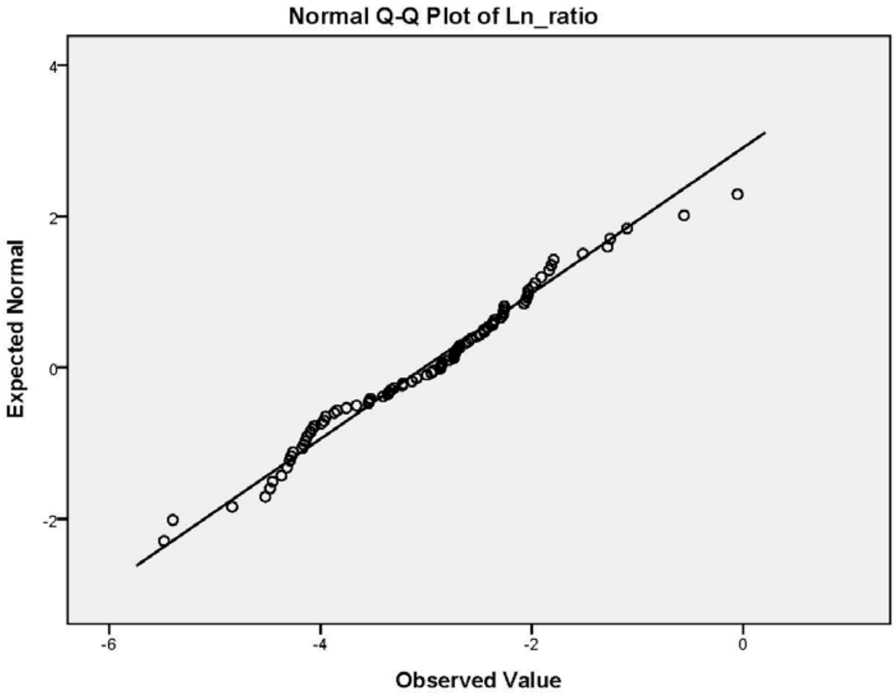

쪽 2

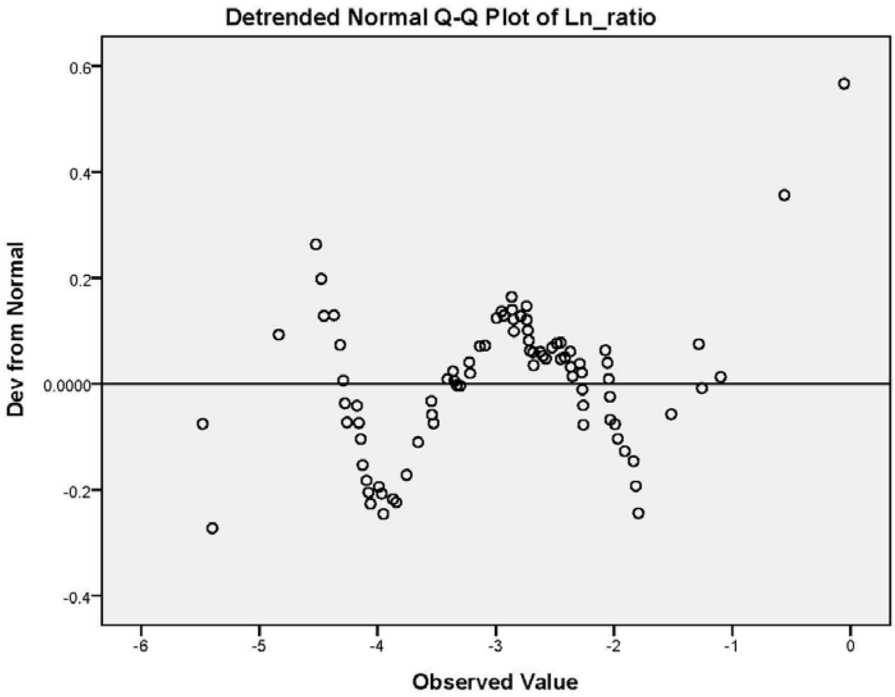
Detrended Normal Q-Q Plot of Ln_ratio
Dev from Normal
0.6
0.4
0.2
0.0000
-0.2
-0.4
-6
-5
-4
-3
-2
-1
0
Observed Value

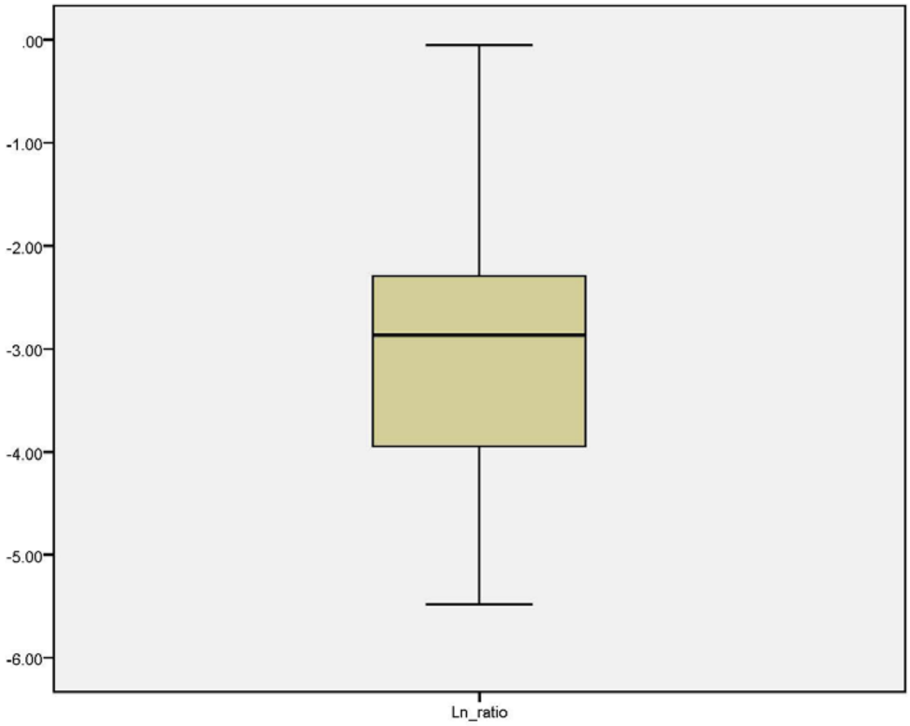


```
ONEWAY Ln_ratio BY Job_5
  /STATISTICS HOMOGENEITY WELCH
  /MISSING ANALYSIS
  /POSTHOC=TUKEY ALPHA(0.05).
```

Oneway

Test of Homogeneity of Variances

Ln_ratio

Levene Statistic	df1	df2	Sig.
1.682	4	85	.162

쪽 4

ANOVA

Ln_ratio

	Sum of Squares	df	Mean Square	F	Sig.
Between Groups	15.681	4	3.920	4.146	.004
Within Groups	80.371	85	.946		
Total	96.052	89			

Robust Tests of Equality of Means

Ln_ratio

	Statistic[a]	df1	df2	Sig.
Welch	6.893	4	18.116	.001

a. Asymptotically F distributed.

Post Hoc Tests

Multiple Comparisons

Dependent Variable: Ln_ratio

Tukey HSD

					95% Confidence Interval	
(I) Job_5	(J) Job_5	Mean Difference (I-J)	Std. Error	Sig.	Lower Bound	Upper Bound
Collector	Driver	.42579	.35717	.756	-.5697	1.4213
	St.sweeper	.33321	.25591	.691	-.3801	1.0465
	Blower	-.92509*	.30862	.029	-1.7853	-.0649
	CBA.collector	.34812	.50882	.959	-1.0701	1.7663
Driver	Collector	-.42579	.35717	.756	-1.4213	.5697
	St.sweeper	-.09258	.38476	.999	-1.1650	.9798
	Blower	-1.35089*	.42166	.016	-2.5261	-.1756
	CBA.collector	-.07767	.58433	1.000	-1.7063	1.5510
St.sweeper	Collector	-.33321	.25591	.691	-1.0465	.3801
	Driver	.09258	.38476	.999	-.9798	1.1650
	Blower	-1.25831*	.34017	.003	-2.2064	-.3102
	CBA.collector	.01491	.52855	1.000	-1.4583	1.4881
Blower	Collector	.92509*	.30862	.029	.0649	1.7853
	Driver	1.35089*	.42166	.016	.1756	2.5261
	St.sweeper	1.25831*	.34017	.003	.3102	2.2064
	CBA.collector	1.27322	.55598	.158	-.2764	2.8229

쪽 5

Multiple Comparisons

Dependent Variable: Ln_ratio

Tukey HSD

(I) Job_5	(J) Job_5	Mean Difference (I-J)	Std. Error	Sig.	95% Confidence Interval	
					Lower Bound	Upper Bound
CBA.collector	Collector	-.34812	.50882	.959	-1.7663	1.0701
	Driver	.07767	.58433	1.000	-1.5510	1.7063
	St.sweeper	-.01491	.52855	1.000	-1.4881	1.4583
	Blower	-1.27322	.55598	.158	-2.8229	.2764

*. The mean difference is significant at the 0.05 level.

Homogeneous Subsets

Ln_ratio

Tukey HSD[a,b]

Job_5	N	Subset for alpha = 0.05	
		1	2
Driver	9	-3.4421	
CBA.collector	4	-3.3644	
St.sweeper	22	-3.3495	
Collector	42	-3.0163	-3.0163
Blower	13		-2.0912
Sig.		.867	.225

Means for groups in homogeneous subsets are displayed.

a. Uses Harmonic Mean Sample Size = 9.856.

b. The group sizes are unequal. The harmonic mean of the group sizes is used. Type I error levels are not guaranteed.

```
ONEWAY Ln_ratio BY Season
  /STATISTICS HOMOGENEITY WELCH
  /MISSING ANALYSIS
  /POSTHOC=TUKEY ALPHA(0.05).
```

Oneway

Test of Homogeneity of Variances

Ln_ratio

Levene Statistic	df1	df2	Sig.
4.022	2	87	.021

ANOVA

Ln_ratio

	Sum of Squares	df	Mean Square	F	Sig.
Between Groups	25.688	2	12.844	15.880	.000
Within Groups	70.365	87	.809		
Total	96.052	89			

Robust Tests of Equality of Means

Ln_ratio

	Statistic[a]	df1	df2	Sig.
Welch	14.244	2	50.449	.000

a. Asymptotically F distributed.

Post Hoc Tests

Multiple Comparisons

Dependent Variable: Ln_ratio

Tukey HSD

(I) Season	(J) Season	Mean Difference (I-J)	Std. Error	Sig.	95% Confidence Interval	
					Lower Bound	Upper Bound
Spring	Autumn	-1.16689*	.20940	.000	-1.6662	-.6676
	winter	-.76025*	.26700	.015	-1.3969	-.1236
Autumn	Spring	1.16689*	.20940	.000	.6676	1.6662
	winter	.40664	.27140	.297	-.2405	1.0538
winter	Spring	.76025*	.26700	.015	.1236	1.3969
	Autumn	-.40664	.27140	.297	-1.0538	.2405

*. The mean difference is significant at the 0.05 level.

Homogeneous Subsets

쪽 7

Ln_ratio

Tukey HSD[a,b]

Season	N	Subset for alpha = 0.05	
		1	2
Spring	39	-3.6111	
winter	16		-2.8509
Autumn	35		-2.4442
Sig.		1.000	.242

Means for groups in homogeneous subsets are displayed.

a. Uses Harmonic Mean Sample Size = 25.704.

b. The group sizes are unequal. The harmonic mean of the group sizes is used. Type I error levels are not guaranteed.

```
ONEWAY Ln_ratio BY Employment
  /STATISTICS HOMOGENEITY WELCH
  /MISSING ANALYSIS
  /POSTHOC=TUKEY ALPHA(0.05).
```

Oneway

Warnings

Post hoc tests are not performed for Ln_ratio because there are fewer than three groups.

Test of Homogeneity of Variances

Ln_ratio

Levene Statistic	df1	df2	Sig.
8.755	1	88	.004

ANOVA

Ln_ratio

	Sum of Squares	df	Mean Square	F	Sig.
Between Groups	16.402	1	16.402	18.121	.000
Within Groups	79.651	88	.905		
Total	96.052	89			

Robust Tests of Equality of Means

Ln_ratio

	Statistic[a]	df1	df2	Sig.
Welch	18.383	1	81.719	.000

a. Asymptotically F distributed.

```
ONEWAY Ln_ratio BY City_2
  /STATISTICS HOMOGENEITY WELCH
  /MISSING ANALYSIS
  /POSTHOC=TUKEY ALPHA(0.05).
```

Oneway

Warnings

Post hoc tests are not performed for Ln_ratio because there are fewer than three groups.

Test of Homogeneity of Variances

Ln_ratio

Levene Statistic	df1	df2	Sig.
4.689	1	88	.033

ANOVA

Ln_ratio

	Sum of Squares	df	Mean Square	F	Sig.
Between Groups	18.198	1	18.198	20.570	.000
Within Groups	77.854	88	.885		
Total	96.052	89			

Robust Tests of Equality of Means

Ln_ratio

	Statistic[a]	df1	df2	Sig.
Welch	21.040	1	85.689	.000

a. Asymptotically F distributed.
